# Cannabinoid CB_1_ and CB_2_ Receptor Signaling and Bias

**DOI:** 10.1089/can.2016.0037

**Published:** 2017-03-01

**Authors:** Mikkel Søes Ibsen, Mark Connor, Michelle Glass

**Affiliations:** ^1^ Department of Pharmacology, School of Medical Sciences, University of Auckland, Auckland, New Zealand.; ^2^ Department of Biomedical Sciences, Faculty of Medicine and Health Sciences, 2 Technology Place, Macquarie University, New South Wales, Australia.

**Keywords:** agonist bias, cannabinoid receptors, functional selectivity, G protein-coupled receptor

## Abstract

An agonist that acts through a single receptor can activate numerous signaling pathways. Recent studies have suggested that different ligands can differentially activate these pathways by stabilizing a limited range of receptor conformations, which in turn preferentially drive different downstream signaling cascades. This concept, termed “biased signaling” represents an exciting therapeutic opportunity to target specific pathways that elicit only desired effects, while avoiding undesired effects mediated by different signaling cascades. The cannabinoid receptors CB_1_ and CB_2_ each activate multiple pathways, and evidence is emerging for bias within these pathways. This review will summarize the current evidence for biased signaling through cannabinoid receptor subtypes CB_1_ and CB_2_.

## Introduction

**FIG. 1.**
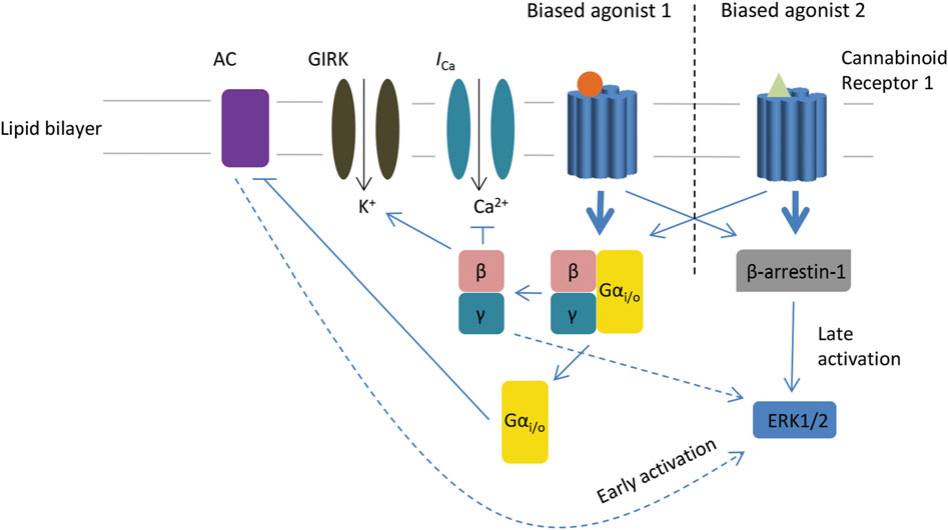
Biased agonist 1 or 2 binds to the seven-transmembrane cannabinoid receptor 1. Structurally different ligands will induce diverse conformations of the receptor, which may then favor one of the possible signaling pathways over others. In this diagram, agonist 1 is biased toward the activation of the Gα_i/o_ heterotrimer over β-arrestin-1, while agonist 2 favorably activates β-arrestin-1. Activation of Gα_i/o_ prompts the release of the Gβγ subunit, which inhibits voltage-dependent calcium channels (*I*_Ca_) and activates GIRK. The Gα_i/o_ subunit inhibits AC, which stimulates the phosphorylation and early activation of ERK1/2. The activation of β-arrestin-1 conversely induces late activation of ERK1/2. AC, adenylyl cyclase; ERK1/2, extracellular signal-regulated kinase 1 or 2; GIRK, G protein-gated inwardly rectifying potassium channels.

Identifying and characterizing the molecular determinants of agonist efficacy in signaling pathway activation are a vital requisite of contemporary drug design. One of the determinants of agonist efficacy is the molecular structure of the agonist, and thus the receptor conformation that it induces. However, receptor conformation is also affected by interactions with various intracellular signaling proteins.^1–3^ For example, the conformation of the β2 adrenergic receptor has been demonstrated to be quite distinct in the presence of the second messenger protein G_s_.^2,3^ Receptor activation will therefore be both ligand and tissue specific, as the assortment and abundance of intracellular signaling constituents vary between cell types. Biased signaling is the concept that different ligands acting on the same G protein-coupled receptor (GPCR), in the same tissue, can give rise to markedly different cellular responses (Fig. 1), and this is likely due to each ligand stabilizing different receptor conformations. This concept has been given many different names—“stimulus trafficking,” “functional selectivity,” and more recently, “agonist bias” or “biased signaling.” It is important to note that differential signaling pathway activation by different agonists can probably also arise as a consequence of kinetics; if there are significant differences in agonist binding kinetics, the more slowly dissociating ligands may allow receptor conformations that favor low-affinity interactions for a particular receptor/signaling molecule pair to persist long enough for productive coupling.

Traditional approaches to demonstrating bias have focused on comparisons of EC50 or Emax values within different pathways, but such methods may not account for inherent differences between pathway stoichiometry. For example, some pathways may achieve maximum response at lower receptor occupancy, resulting in higher potency for all agonists within this pathway (pathway bias). Quantifying biased signaling involves determining the effects of two or more agonists on two or more cellular responses, and comparing the agonist profiles for each pathway. More recently, the operational model of bias has been utilized^4^; this approach compares all ligands within each pathway against a reference ligand and then makes comparisons of the relative shifts between pathways relative to the reference.

Therapeutically, it is hoped that the study of biased signaling of GPCR-directed therapeutics will enable engagement of desired pathways over those not involved in the therapeutic effect. Ideally, this would eliminate on-target but unwanted or adverse effects; agonists or allosteric modulators that induce signaling in a biased manner could potentially revolutionize future therapeutic drugs. The μ-opioid receptor presents a prime example where biased signaling might be exploited at the translational level. Although opioid receptors are useful targets of analgesics, μ-opioid receptor activation also causes respiratory depression, which is suggested to be a product of β-arrestin2 recruitment.^5^ Hence, the development of an agonist that preserves the analgesic (G protein-mediated) properties of activated μ-opioid receptors without any respiratory side effects would be beneficial to avoid adverse effects, and such compounds have recently been developed in light of this hypothesis.^6,7^

The cannabinoid receptor family consists of two GPCRs, cannabinoid receptor 1 (CB_1_) and cannabinoid receptor 2 (CB_2_), and they will be the focus of this review. CB_1_ plays a role in regulating neurotransmission in many brain regions. When activated, CB_2_ regulates immune responses and inflammatory pathways. Mice lacking CB_2_ often demonstrate an exacerbated inflammatory phenotype^8^ and besides roles in the periphery expression in brain microglia suggest a role for CB_2_ in neuroinflammation.^9,10^ Recently, CB2 has been suggested to contribute to neuronal plasticity in mouse hippocampal neurons^11^ potentially expanding the role of this receptor in the brain. The endogenous lipids anandamide and 2-arachidonoylglycerol (2-AG) are the physiological cannabinoid receptor agonists and are hence known as endocannabinoids.^12–14^ A great deal of interest has centered on the potential role of CB_1_ in targeting a range of central nervous system (CNS) disorders such as pain,^15^ anxiety,^16^ multiple sclerosis,^17^ obesity,^18^ nicotine addiction,^19^ Huntington disease,^20^ and Parkinson's disease.^21^ In more recent times CB_2_ has also become a focus in peripheral inflammatory disorders such as nephrotoxicity.^8,22^

In addition to the two well-established cannabinoid receptors, several other GPCRs have been reported to be activated by cannabinoid drugs or endocannabinoids and related molecules, including GPR55,^23^ GPR18,^24^ and GPR119.^25^ Furthermore, endogenous and synthetic cannabinoids can activate and potentiate transient receptor potential (TRP) channels^26^ and glycine receptors,^27^ respectively. The potential contribution of these receptors to the therapeutic effects of cannabinoids or the physiological effects of endocannabinoids are only beginning to be explored, and much less is known about how ligands regulate their signaling.

The widespread distribution of CB_1_ in the CNS provides a strong rationale for developing ligands with biased profiles to potentially avoid the consequences of activating multiple signaling pathways in many different brain regions. Biased ligands could also potentially have context-dependent effects, providing effective modulation of pathways dysregulated by disease in restricted subsets of neurons. Modulating selected downstream pathways would result in a more targeted pharmacological response, but considerable research is still required to understand which of the characterized pathways are therapeutically desirable, and under which disease condition.

## Signaling of Cannabinoid Receptors

### G protein coupling of cannabinoid receptors

First cloned in the early 1990s,^28,29^ both CB_1_ and CB_2_ were initially described as exhibiting pertussis toxin-sensitive signaling through Gα_i/o_-type G proteins,^30^ however, differences in signaling were reported from the outset. When expressed in AtT-20 cells, CB_1_ mediated an inhibition of P/Q-type calcium channels and an activation of G protein-gated inwardly rectifying potassium channels (GIRKs) in addition to inhibition of adenylyl cyclase.^30^ By contrast, CB_2_ was not reported to modulate the activity of either channel in AtT-20 cells but did inhibit adenylyl cyclase.^30^ This poor coupling may be an example of functional selectivity as it was recently found that mouse CB_2_ (mCB_2_) can inhibit voltage-gated calcium channels, in a manner strongly dependent on the CB_2_ ligand used, with CP55,940, but not WIN55,212-2, able to inhibit the channels via a Gβγ pathway.^31^ It is unclear how far this apparent biased signaling extends, however, as both WIN55,212-2 and CP55,940 hyperpolarize AtT-20 cells expressing human CB_2_ (hCB_2_), and this response almost certainly reflects Gβγ subunit-mediated activation of GIRKs.^32^

In studies using purified G proteins, Glass and Northup^33^ demonstrated that while CB_1_ and CB_2_ had similar affinity for Gα_i_-type G proteins, CB_2_ had significantly lower affinity for Gα_o_ than Gα_i_. CB_1_ is promiscuous in its G protein coupling, with activation of Gα_s_- and Gα_q_-dependent signaling under some conditions, or in some cells. Gα_s_-like coupling has been suggested under circumstances where Gα_i_ activation is limited, such as following pertussis toxin treatment, or simultaneous activation of CB_1_ with other Gα_i_ linked receptors such as D2^34–36^; indeed it appears that in some cell types the Gα_s_ linkage is dominant.^37,38^ Studies have suggested that the second intracellular loop of CB_1_ mediates both Gα_s_ and Gα_i_ coupling specificity,^39^ but a detailed understanding of what regulates the specific G protein coupling is still lacking. Gα_q_ coupling of CB_1_ has been suggested for only a few ligands^40^ and will be discussed in more detail below. Finally, *in vivo* studies have also suggested coupling of CB_1_ to the Gα_i/o_-related G protein Gα_z_.^41^ The ability to activate such a diverse range of G proteins strongly suggests that biased signaling between different G protein pathways could be achieved, and examples of this are emerging and described below. Apart from the interactions with Gα_i_ and Gα_o_ less is known about additional G proteins interacting with CB_2_.

The consequences of activating Gα_i_, Gα_o_, Gα_s_, and Gα_q_ heterotrimers have been described for many GPCRs, and this signaling seems similar for the most part for CB_1_ and CB_2_. Gα_i/o_ subunits inhibit adenylyl cyclase or couple to the mitogen-activated protein kinase (MAPK) pathway. Gα_s_ stimulates adenylyl cyclase (and subsequent phosphorylation of cAMP response element-binding protein [CREB]). Gα_q_ couples to phospholipase C and promotes release of intracellular calcium ([Ca]_i_). Gβγ subunits derived from Gα_i/o_ activate GIRKs (comprising K_IR_ 3.X heteromers), activate phosphatidylinositide-3-kinase, and inhibit voltage-dependent calcium channels (*I*_Ca_).^42–44^

Originally described as “G protein-coupled” proteins based on their modulation of classical signaling pathways via heterotrimeric G proteins, GPCRs also recruit other proteins for signaling, most prominently β-arrestin-1 and -2. Arrestins serve multiple functions as regulators of G protein coupling and receptor localization, and are essential elements of multiple GPCR signaling cascades involving kinases, phosphatases, and ubiquitin ligases.^45^ Arrestin signaling has been generally thought to occur in the absence of bound G protein, and although this has been recently challenged,^46^ arrestin-mediated signaling is likely to be mediated by a different ligand/receptor conformation from those involved in Gα interactions.

### Activation of MAPK cascades

MAPK family members have been found to regulate diverse biological functions by phosphorylation of specific target molecules (such as transcription factors) and thereby participate in the regulation of a variety of cellular processes, including cell proliferation, differentiation, and apoptosis.^47^

As for most GPCRs, there are multiple pathways by which CB_1_ activation can lead to phosphorylation of extracellular signal-regulated kinase 1 or 2 (ERK1/2). In Chinese hamster ovary (CHO), U373 MG, and PC-3 cells, phosphatidylinositide 3-kinase activation is required for CB_1_ activation of ERK1/2, and the Gβγ subunit, rather than Gα, may transduce the CB_1_ signal.^48,49^ In contrast to this, ERK1/2 activation in N1E-115 mouse neuroblastoma cells, or mouse hippocampal slices, is reported to be downstream of inhibition of cyclic adenosine monophosphate (cAMP)/protein kinase A.^50,51^

CB_1_ may also transactivate members of the receptor tyrosine kinase family leading to subsequent activation of ERK1/2. Several tyrosine kinase receptors have been implicated in this pathway, including the vascular endothelial growth factor receptor^52^ and epidermal growth factor receptor.^53^ The Src-family kinase, Fyn, is also likely to be involved in the activation of ERK1/2 by the CB_1_-transactivated tyrosine kinase receptors, as Fyn knockout (KO) mice show no elevation of phosphorylated ERK1/2 (pERK1/2) in response to cannabinoid administration.^51^

Regardless of the precise pathway mediating pERK1/2 downstream of CB_1_ activation, all of the above studies found the activation to be downstream of Gα_i/o_ protein signaling (and therefore pertussis toxin sensitive), however, pertussis toxin-insensitive ERK1/2 activation has also been demonstrated in rat CB (rCB_1_)-transfected human embryonic kidney 293 (HEK293) cells.^54^ The Gα_i/o_ protein-independent pERK1/2 activation might arise from the recruitment of β-arrestin-1 as mutation of phosphorylation sites in the CB_1_ C-terminal (S426A/S430A) has been suggested to result in β-arrestin-1-mediated pERK activation,^55^ which in general occurs later than the G protein-mediated activation.^56^ Similarly, as discussed below, ORG27569, an allosteric modulator of CB_1_, has been suggested by some studies^57,58^ but not others^59,60^ to activate β-arrestin-1-mediated pERK1/2 signaling through CB_1_.

Activation of other MAPKs downstream of CB_1_ has been reported, although there is considerable divergence between tissues and ligands used among the studies, which makes reconciling contrasting results difficult. In rat hippocampal slices, anandamide, CP55,940, WIN55,212-2, and Δ^9^-tetrahydrocannabinol (THC) activate p38 MAPK, but not c-Jun N-terminal kinase (JNK).^61^ In cultured cortical neurons, JNK was activated when stimulating with THC in a pertussis toxin-sensitive manner,^62^ however, HU-210 failed to stimulate ERK1/2, p38 MAPK, or JNK in a subsequent study on cultured hippocampal neurons.^63^ In Neuro-2a cells endogenously expressing mCB_1_, HU-210 activated only ERK1/2, but not JNK or p38 MAPK pathways,^64^ although a different study found that HU-210 stimulated JNK activation in these cells.^65^ Rueda et al.^66^ showed that THC-mediated activation of JNK was dependent on Gα_i/o_ proteins, phosphatidylinositide 3-kinase, and Ras, and involved platelet-derived growth factor (PDGF) receptor transactivation in CHO cells. In contrast, in the same cells, THC activation of p38 MAPK was not dependent on PDGF receptor activation,^66^ suggesting the possibility of multiple CB_1_-stimulated pathways for kinase activation in the same cells.

There are relatively few studies on CB_2_ activation of MAPK pathways. While several studies have investigated the ability of cannabinoid agonists to modulate ERK1/2 activation in response to inflammatory stimuli,^67,68^ only one study has examined the pathway by which CB_2_ may link to ERK activation. In this study, hCB_2_ coupling to ERK1/2 was shown to be pertussis toxin sensitive but independent of cAMP.^69^ Some of the variability observed in the pathways activating kinases downstream of both CB_1_ and CB_2_ may reflect different levels of receptor expression between cells as previous studies have demonstrated that the ability to activate some kinases (most notably pAkt) required high receptor expression, whereas ERK1/2 activation occurred at all levels of expression.^70^

### Desensitization, arrestin recruitment, and signaling

A common pathway for uncoupling GPCRs from G protein-dependent signaling is receptor phosphorylation followed by binding of an arrestin.^71^ In this sequence of events, G protein receptor kinases (GRKs) have a prominent role phosphorylating the GPCR after agonist binding, and this phosphorylation increases the affinity of arrestin for the receptor. Arrestin binds to domains of the receptor thought to interact with G proteins, preventing receptor activation of these effectors. It is also possible that phosphorylation of a receptor before arrestin binding disrupts receptor/G protein interactions. The ultimate consequences of arrestin binding are complex; for many receptors, arrestin binding is a step in a pathway that leads to removal of the receptor from the plasma membrane, however, arrestin also acts as a scaffolding molecule for non-G protein-mediated signaling pathways such as activation of ERK.

In a classic series of studies, Howlett et al. delineated CB_1_-mediated inhibition of agonist-stimulated adenylyl cyclase activity in N18TG2 cells.^72^ This work included showing that desacetyllevonantradol (CP54,939) inhibition of adenylyl cyclase maximally desensitized within 30 min of drug exposure.^72^ The desensitization of CB_1_ responses was homologous, as it did not affect the muscarinic receptor-mediated inhibition of adenylyl cyclase and did not affect the maximum accumulation of cAMP produced by the stimulatory agonist, secretin.^73^ THC also produced desensitization in this assay, but it was slower and less complete than that produced by CP54,939. Similar effects of CP55,940 on hCB_1_ expressed in CHO cells indicate that rapid desensitization of CB_1_ coupling to adenylyl cyclase is common.^74^ While these assays provide a good indication that receptor desensitization is rapid, interpretation of the quantitative elements of the studies is difficult as the assays utilized 20 min or more of agonist exposure to define control responses, and this period almost certainly encompasses many significant regulatory events.

By contrast to traditional single-point assays of cAMP accumulation or ERK phosphorylation, GPCR modulation of ion channels provides a continuous and relatively direct readout of receptor activity, particularly when Gβγ inhibition of *I*_Ca_ or activation of GIRK is measured.^75^ Unfortunately, there are very few cell lines where native CB_1_ receptors coexist with voltage-gated *I*_Ca_ or GIRK. NG-108-15 and N18 cells, with endogenous CB_1_, were used for the very earliest descriptions of CB_1_ receptor inhibition of *I*_Ca_,^76–78^ however, receptor regulation was not examined. Recombinant CB_1_ receptors expressed in murine AtT-20 cells couple to both inhibition of *I*_Ca_ and activation of GIRK,^79^ and desensitization of GIRK activation has been reported.^80^ AtT-20 cells and *Xenopus* oocytes have been used to provide some insight into CB_1_ regulation of ion channels.^80,81^ In oocytes, desensitization of rCB_1_-mediated activation of GIRK was shown to be stimulated by coexpression of both GRK3 and β-arrestin-2, but not affected by either protein alone.^80^ The efficiency of other members of the GRK family, or of β-arrestin-1, was not addressed in the oocyte studies, nor were the effects of coexpression of these molecules on basal coupling of CB_1_ to GIRK.

Mutation of two of six available serine/threonine residues in the C-terminal tail (S426/S430) of rCB_1_ was sufficient to block GRK3/β-arrestin-2-mediated desensitization of coupling to GIRK in oocytes.^80^ When CB_1_ missing the last 55 intracellular residues was expressed in AtT-20 cells, WIN55,212-2 desensitization was abolished suggesting a role for the putative GRK phosphorylation sites contained in the missing domain in desensitization. Unfortunately, coupling of the S426A/S430A mutant to native GIRK in AtT-20 cells was not measured, and the role of GRK3 (or other GRK family members) in regulation of CB_1_ in these cells has not been directly addressed.

Direct recruitment of β-arrestin-2 to activated CB_1_ has also been demonstrated in both AtT20 and HEK293 cells.^54,82^ In a detailed study of C-terminal mutants, Daigle et al.^82^ found that all internalization-competent CB_1_ mutants could recruit β-arrestin-2, although some mutants appeared to recruit β-arrestin-2 at a reduced rate, while mutation of all six serine/threonine residues in the rCB_1_ C-terminus (460–473) prevented internalization and also failed to recruit β-arrestin-2. A recent detailed bioluminescence resonance energy transfer (BRET)-based study of CB_1_ arrestin interactions, suggested a low-affinity, transient interaction between CB_1_ and β-arrestin-2, with no interaction in late endosomes consistent with a family-A interaction, while recruitment of β-arrestin-1 by orthosteric ligands was not observed.^83^ Structural studies have suggested an interaction between β-arrestin-1 with a synthesized CB_1_ C-terminus,^84,85^ and this has recently been observed in a whole cell.^86^

β-arrestin-2 KO mice revealed a role of arrestin in CB_1_-mediated signaling. THC produced both greater antinociception and greater decreases in body temperature in β-arrestin-2 KO mice compared with wild-type mice, consistent with a role for arrestins in blunting receptor signaling, however, the action of a range of synthetic ligands was normal.^87^ Tolerance to THC antinociceptive effects was also reduced in KO mice and decreased downregulation of CB_1_ was observed.^88^ These studies could suggest substantial agonist differences in arrestin recruitment for different assays, which requires further study. Alternatively, as THC is a partial agonist at CB_1_, it may be more sensitive to subtle changes in receptor availability as presumably it requires full occupancy to exert maximum effect. Finally, as with all studies of global KO animals, it is possible that the signaling of many GPCRs in the circuits that mediate cannabinoid effects is altered, and has been since the start of the animals' life. Single-cell studies examining CB_1_ receptor function and regulation have not be done using cells from arrestin KO animals. Studies on Gα_i/o_-coupled μ-opioid receptors in single neurons from KO animals show that there is not always a clear correlation between changes in agonist actions at these neurons and with changes in behavior.^89,90^

Surprisingly, there has been little detailed study of CB_2_-mediated arrestin recruitment. McGuinness et al.^91^ and Dhopeshwarkar and Mackie^92^ utilized the PathHunter DiscoveRx assay to investigate the ability of a range of cannabinoid ligands to recruit β-arrestin-2 at hCB_2_ and mCB_2_, respectively. This assay utilizes enzyme complementation to detect recruitment of tagged arrestin to the receptor. McGuinness et al. observed robust and potent recruitment of arrestin to a range of cannabinoid ligands, including CP55,940, JWH015, and WIN55,212-2.^91^ Dhopeshwarkar and Mackie found that nonclassic ligands (CP55,940) recruited arrestin, whereas classic cannabinoid ligands (THC, JWH133, KM233, etc.) did not, suggesting a strong functional bias with structure.^92^ Recently, Soethoudt et al.^32^ also investigated the ability of a wide range of ligands to activate arrestin utilizing this assay and found that all ligands modulated arrestin recruitment to some extent. CP55,940 was the most potent ligand in this assay, followed by WIN55,212-2. Only CP55,940 acted as full agonist, while WIN55,212-2, JWH133, JWH015, HU-308, HU-910 were partial (Emax 50–70%) and the endocannabinoids (Emax 40–80%) only partially recruited arrestin.^32^ Interestingly, JWH133, HU-308, and HU-910 were all significantly less potent on mCB_2_ than hCB_2_ in arrestin recruitment, strongly suggesting species differences. A potential confounding issue with all these assays is the protein modules added to the receptor and arrestin constructs to enable visualization of the interactions using optical techniques. The signaling capacity of these constructs is not usually assessed, and so it is not obvious that the conformational changes being reported faithfully reflect those of the native receptor and effector. In summary, while there is some evidence to suggest that arrestin-biased signaling is observed between cannabinoid ligands, the current lack of consistency between studies needs to be resolved to enable the design of genuinely biased ligands.

### Post-endocytic regulation of cannabinoid receptors

CB_1_ undergoes rapid internalization following agonist stimulation.^93,94^ While early studies suggested that CB_1_ may recycle following internalization,^94,95^ subsequent detailed studies have suggested that the internalized receptor enters degradative pathways^96^ and is rapidly degraded, with resensitization requiring the delivery of newly synthesized receptors.^93^

Internalization of CB_1_ has generally been shown to be clathrin mediated,^94,97^ although in some systems, caveolae-mediated internalization has been observed.^97^ Mutation of a highly conserved aspartate residue in the second intracellular loop (D164 in rCB_1_) resulted in a loss of CB_1_ internalization in AtT20 cells.^81^ Intriguingly, receptors with this mutation demonstrated preserved binding, cAMP inhibition, and inhibition of Ca^2+^ currents but did not activate GIRK.^81^ This mutation has also been suggested to decrease the constitutive activity of CB_1_.^98^ Further studies identified the extreme carboxy-terminal tail of the rCB_1_ as a central mediator of agonist-induced internalization of the receptor in AtT20 cells.^94^ Subsequently, truncation of this region was also shown to prevent arrestin recruitment to CB_1_ in AtT20 cells.^82^ However, the truncated receptor internalized normally when expressed in HEK293 cells.^82^ Mutation of all of the serine/threonine residues in this region resulted in failure to recruit arrestin and a decreased extent of internalization, although the initial rates were normal.^82^ Changes in the extent of internalization are challenging to interpret, as higher expression levels frequently result in a decreased extent of internalization of receptors in transfected cell lines, likely due to saturation of the internalization machinery. Thus, the precise mechanisms controlling internalization kinetics are far from clear.

Studies on CB_1_ trafficking have been complicated by the presence of a large intracellular pool of receptors observed in both primary and recombinant cell lines, and native expressing tissues.^93^ While it was predicted that these receptors represented a pool of receptors from which CB_1_ could be rapidly mobilized to the cell surface,^95^ mobilization has not yet been observed, although the receptors do undergo constitutive synthesis and degradation.^93^ Recent studies have suggested that these intracellular receptors may be involved in signaling,^99,100^ a suggestion made more feasible by the high lipid solubility of the endocannabinoid ligands. Intracellular CB_1_ has recently been suggested to be present in mitochondria in neurons and astrocytes, suggested to signal through mitochondrial G proteins leading to inhibition of cAMP and mitochondrial respiration.^101,102^

Studies detailing the trafficking properties of CB_2_ are limited. Early studies utilizing an antibody that failed to detect hCB_2_ when phosphorylated at serine 352 suggested that CB_2_ undergoes agonist-mediated phosphorylation in response to CP55,940. The phosphorylation was accompanied by decreased signaling in both CHO and HL60 cells.^103,104^ Agonist-mediated internalization has also been observed in response to CP55,940^103,105^ and 2-AG.^103,106^ Early studies suggested that CB_2_ was repeatedly phosphorylated and dephosphorylated by alternate stimulation with agonist and inverse agonist implying that CB_2_ recycling may occur,^69^ and this was more recently demonstrated to be the case, and to be Rab11 dependent.^107^ The effect of the inverse agonist SR144528 on CB_2_ internalization is unclear as some find a decrease in cell surface CB_2_,^103^ while others find no change.^106^ One recent study has suggested that chronic treatment with a cannabinoid agonist leads to CB_2_-mediated upregulation of GRK5 in rat prefrontal cortex via a β-arrestin-2-dependent pathway,^108^ but further molecular studies are required.

For many receptors, bias has been observed in the ability of ligands to induce internalization.^109^ The consequences of such bias are not straightforward to interpret. We can speculate that if a receptor remained on the cell surface longer in the presence of a particular ligand, it might exhibit prolonged signaling, however, equally, if the receptors are desensitized but remain on the cell surface, feedback mechanisms governing the recycling of the receptor (for CB_2_) or delivery of new receptor to the cell surface (for CB_1_) might be altered leading to an overall reduction in signaling.

### Biased signaling of cannabinoid receptors

The diverse chemical structures of commonly used cannabinoid ligands increase the likelihood of identifying biased signaling, and evidence of agonist bias has emerged from a number of studies.

The first evidence for biased signaling through CB_1_ came from assays in which membranes of CB_1_ expressing Sf9 cells, stripped of their endogenous G proteins, were reconstituted with purified G proteins.^33^ These studies demonstrated that the relative activation of Gα_i_ and Gα_o_ is dependent on the agonist. HU-210, WIN55,212-2, and anandamide all elicited maximal Gα_i_ activation, whereas THC caused only partial Gα_i_ activation. In contrast, only HU-210 effected maximal CB_1_ stimulation of Gα_o_, with anandamide, WIN55,212-2, and THC all partially stimulating.^33^ This work was further extended by coimmunoprecipitation of activated G proteins in N18TG2 cells, which demonstrated that WIN55,212-2 behaved as a full agonist for all three Gα_i_ subtypes, while methanandamide appeared to be an agonist at Gα_i3_ and an inverse agonist at Gα_i1_ and Gα_i2_.^110^ In addition, relative signaling efficacies and potencies of CB_1_ ligands differ in different brain regions,^111–113^ which may represent the different G protein compositions of different regions. Intriguingly, plasmon-waveguide resonance (PWR) spectroscopy has demonstrated that CP55,940 and WIN55,212-2 produced distinct spectral changes (PWR shifts in opposite directions) on binding to the hCB_1_ indicating that the two agonists produce qualitatively distinct active conformations of the receptor, which have differing affinity for Gα_i_.^114^ Differential signaling by WIN55,212-2 and CP55,940 is consistent with the suggestion that these ligands have overlapping but distinct binding sites,^115,116^ a finding supported by molecular docking in the recently described crystal structure of CB_1_.^117,118^

Laprairie et al.^20^ investigated the biased signaling of WIN55,212-2, CP55,940, 2-AG, anandamide, THC, cannabidiol, and the combination THC+cannabidiol on several signaling pathways. The agonists were used on *in vitro* medium spiny projection neurons having a wild-type (ST*Hdh*^Q7/Q7^) or Huntington disease (ST*Hdh*^Q111/Q111^) background. The effect on a range of cannabinoid-dependent signaling pathways was measured via pERK1/2 (Gα_i/o_ mediated), β-arrestin-1 recruitment to CB_1_ (by BRET), phosphorylation of CREB (pCREB; suggested to be Gα_s_ mediated), phosphorylation of phospholipase C (pPLCβ3; suggested to be Gα_q_ mediated), and phosphorylation of Akt (pAkt) (Gβγ mediated). The signaling bias was calculated relative to WIN55,212-2 signaling. This study found that CP55,940 induced signaling biased toward Gα_s_ and β-arrestin-1 compared to Gα_i/o_, while Gα_i/o_ signaling was biased compared to Gα_q_ and Gβγ in both cell types (i.e., Gα_s_ > β-arrestin-1 > Gα_i/o_ > Gα_q_ >Gβγ). 2-AG elicited signaling bias toward Gβγ compared to Gα_i/o_ (in ST*Hdh*^Q7/Q7^ cells), and Gα_i/o_-biased signaling compared to β-arrestin-1 (in ST*Hdh*^Q7/Q7^ cells) and Gα_q_ (predominantly in ST*Hdh*^Q111/Q111^) (i.e., Gβγ > Gα_i/o_ > β-arrestin-1 > Gα_q_). Similar to 2-AG, anandamide signaling was biased toward Gβγ compared to Gα_i/o_ (in ST*Hdh*^Q7/Q7^ cells), while Gα_i/o_ signaling was biased compared to β-arrestin-1 and Gα_q_ (mostly in ST*Hdh*^Q111/Q111^) (i.e., Gβγ >Gα_i/o_ > β-arrestin1 > Gα_q_). THC produced signaling biased toward β-arrestin-1, Gα_q_, and Gβγ compared to Gα_i/o_ in both cell types (i.e., β-arrestin-1 >Gα_q_=Gβγ > Gα_i/o_). Since cannabidiol treatment only evoked significant Gα_s_-mediated pCREB, bias values could not be calculated for this ligand. The combination of THC+cannabidiol induced signaling biased toward Gα_s_ compared with Gα_i/o_, while signaling was biased toward Gα_i/o_ compared with β-arrestin-1, Gα_q_, and Gβγ (predominantly in ST*Hdh*^Q7/Q7^ cells) (i.e., Gα_s_ > Gα_i/o_ > β-arrestin1=Gα_q_=Gβγ).

Khajehali et al.^60^ investigated biased signaling and allosteric modulation between cAMP and ERK1/2 activation using CHO cells stably expressing CB_1_ treated with CP55,940, HU-210, WIN55,212-2, THC, methanandamide, anandamide, and 2-AG. pERK1/2 and cAMP levels were measured in response to the treatments. The signaling bias induced by each ligand was calculated relative to 2-AG. CP55,940, HU-210, WIN55,212-2, THC, methanandamide, and anandamide all showed a preference toward cAMP inhibition compared to pERK1/2. HU-210 and methanandamide displayed strong bias toward cAMP inhibition, whereas CP55,940, THC, and anandamide showed nonsignificant bias toward cAMP inhibition. As both these pathways are pertussis toxin sensitive in these cells, this may indicate bias between α- and βγ-mediated pathways (although the exact pathway for pERK1/2 activation was not defined). The mechanism by which α and βγ bias could be mediated is currently unclear.

The putative Gα_s_ coupling of CB_1_ has also shown potential for agonist bias. While equivalent rank order of potencies was observed for inhibiting or stimulating cAMP in CHO-hCB_1_ cells, anandamide and CP55,940 were significantly less efficacious in stimulating the accumulation of cAMP than in inhibiting its formation.^119^ Forskolin acts synergistically with CB_1_-activated Gα_s_ at adenylyl cyclase.^35^ Cannabinoid receptor-mediated stimulation of cAMP also revealed differences among agonists in as much as forskolin enhanced the potency of HU-210 and CP55,940 by ∼100-fold but had no effect on the potency of WIN55,212–2 or anandamide.^119^

Perhaps the most extreme example of agonist bias for one G protein over another through CB_1_ is that observed in the proposed coupling of CB_1_ to Gα_q_. Lauckner et al.^40^ demonstrated that high concentrations of WIN55,212-2, but not THC, HU-210, 2-AG, or methanandamide resulted in increased release of [Ca]_i_ through a Gα_q_ pathway. More recently, rat hippocampal autaptic long-term potentiation has been suggested to be mediated by 2-AG-induced CB_1_ activation of Gα_q_, suggesting that biased signaling may not hold between different cell types.^120^ *N*-arachidonoyl dopamine (NADA) represents an interesting case for a putative highly biased CB_1_ agonist as it is currently only known to affect very select pathways.^121^ NADA is an endocannabinoid agonist of both CB_1_ and TRPV1.^122,123^ NADA binds orthosterically to CB_1_ with high nanomolar affinity but has no substantial effect on GIRK-mediated hyperpolarization, cAMP levels, pERK, or adenylyl cyclase activity. At concentrations above 30 μM, NADA elevates [Ca]_i_ levels from intracellular stores in cell cultures and causes a slow internalization of CB_1_ from the cell surface. The data strongly suggest that NADA signals via a Gα_q_-mediated pathway. Although NADA did not potently induce [Ca]_i_, it has been demonstrated to be more potent under different *in vitro* assay conditions^122^ and in brain slices.^124,125^ In summary, significant bias in activating Gα_q_-mediated responses occurs, with only a small subset of ligands reporting this (WIN55,212-2, NADA, and 2-AG), and then only at high concentrations and in a tissue-dependent manner.

For most GPCRs, interest in biased signaling has focused predominantly on the ability of agonists to differentially modulate G protein and arrestin pathways. The greatest promise for this to date through CB_1_ has come from allosteric modulators. While ORG27569 has been suggested to generally inhibit agonist-mediated G protein activation on its own,^112^ it has been suggested to exhibit biased signaling toward pERK1/2 pathways via β-arrestin-1.^57,58^ This finding is not consistent across all reports, however, as others^59,60^ did not find that treatment with ORG27569 alone induced pERK1/2. The different findings could arise from differences in assay design or differences in the relative receptor expression, and signaling systems between the different cells utilized.^57,58,60,126^ In particular, time-dependent effects on CB_1_ signaling have been reported in several studies of ORG27569 and functionally related molecules,^127,128^ which may account for some variability.

Pregnenolone, a putative endogenous allosteric modulator of CB_1_ function, modifies agonist signaling in a biased or selective manner, inhibiting pERK1/2 signaling while not modifying cAMP signaling.^129^ Interestingly, recent studies have suggested a similar profile for Src homology 3-domain growth factor receptor-bound 2-like (endophilin) interacting protein 1 (SGIP1), a CB_1_ interacting protein expressed in HEK293 cells.^130^ These findings emphasize the challenges in interpreting pERK1/2 studies when the pathway regulating it (G protein versus arrestin mediated) is generally not well defined. An understanding of the conformation of the receptors driven by the presence of allosteric modulators may help guide the development of highly biased agonists as it is likely that a greater range of conformations may be achieved by targeting regions outside of the relatively constrained orthosteric binding site.

Similarly, receptor mutation studies may enhance our understanding of the molecular drivers for bias. A few examples of this are currently emerging, for example, mutations in potential phosphorylation sites in rCB_1_ (S426A/S430A) drive preferential signaling via β-arrestin-1,^55^ while different mutations in the DRY motif drive conformations to preferentially signal through either G proteins or β-arrestins.^131^

Comparatively, there is little evidence for agonist bias through G proteins for CB_2_, although some signaling biases have been described. Shoemaker et al.^105^ compared CP55,940, 2-AG, and 2-arachidonoylglyceryl-ether using MAPK activation, stimulation of calcium transients, and inhibition of adenylyl cyclase as the signaling pathways. Each ligand differed in its rank order of potency in the three assays despite similar efficacies. Schuehly et al.^132^ found that AM630 displayed inverse agonist/antagonist actions on CB_2_-mediated inhibition of cAMP production and was silent in its effects on [Ca]_i_ transients. On the contrary, a novel CB_2_ ligand, 4-*O*-methylhonokiol, was an inverse agonist/antagonist with regard to cAMP production but potentiated the effects of 2-AG on calcium transients. Atwood et al.^31^ have further demonstrated that CP55,940, but not WIN55,212-2, leads to inhibition of voltage-gated calcium channels through CB_2_, in addition to different actions on receptor trafficking.

Recent studies have suggested agonist bias in CB_2_ internalization. Atwood et al.^31^ found that while CP55,940 induced robust internalization of rCB_2_, WIN55,212-2 failed to promote receptor internalization, despite both agonists activating pERK, and arrestin recruitment. Extending these studies, Dhopeshwarkar and Mackie^92^ examined a range of ligands in inhibiting adenylyl cyclase, internalization, and arrestin recruitment to mCB_2_. Of the most commonly utilized ligands, CP55,940 and JWH015 were the most balanced compounds evaluated although JWH015 had lower efficacy. The majority of the other compounds screened were G protein biased, while a few less commonly utilized ligands (STS135; UR144; 4-*O*-methylhonokiol; and GW833972A) were more arrestin biased. It will be interesting to see if this difference results in different tolerance profiles to these agonists in CB_2_-mediated effects.

In a highly detailed analysis of functional selectivity of CB_2_, Soethoudt et al.^32^ recently analyzed the ability of a wide range of ligands to signal through hCB_2_ and analyzed the data with operational analyses. This analysis suggested that THC showed bias toward pERK signaling compared to arrestin and GTPγS, and intriguingly, THC did not activate GIRK, indicative of high bias against this pathway. (R,S)-AM1241 was biased toward arrestin coupling and pERK signaling compared to GIRK channel activation. JWH133 was moderately biased toward arrestin compared to GIRK, whereas both WIN55,212-2 and JWH015 showed preference for GIRK compared to cAMP signaling. Anandamide showed preference for pERK and GIRK signaling compared to cAMP, whereas 2-AG was significantly biased toward GIRK compared to cAMP and G protein signaling. On comparison between arrestin coupling and cAMP signaling, all ligands appear to be significantly biased, however, this may reflect the choice of CP55,940 as the reference ligand, as this ligand appears itself biased toward cAMP signaling. The authors of this study concluded that HU-910 and HU-308 were well-balanced ligands without significant bias toward any signal transduction pathway on hCB_2_, but this study also highlighted species differences, as at mCB_2_, HU-910 and HU-308 were significantly biased toward G protein signaling over arrestin coupling. A limitation of this study is that the different assays were conducted in different cells lines, and thus, some differences may represent different expressions of signaling molecules (e.g., different βγ-subunit expression), which requires further investigation. The study concludes that THC, 2-AG, and (R,S)-AM1241 are highly biased CB_2_ agonists and underscores that biased signaling at CB_2_ is subject to species variation.

## Conclusions

The concept of agonist bias provides an exciting new direction for developing therapeutics with less adverse effects. However, for the cannabinoid receptors, this field is still in its infancy. The influence of different cell types, with different receptor numbers and second-messenger expressions in the signaling pathways measured, remains to be fully elucidated. This review highlights that CB_1_- and CB_2_-biased signaling can be different between tissues and species, which might prove an issue for translating results from *in vitro* studies to *in vivo*. As a large body of the work describing biased signaling of the cannabinoid receptors is performed using heterologously expressed receptors, future work should include endogenously expressed receptors to determine if previous observations are relevant.

Importantly, almost all of the studies described here measured single time points for each signaling assay, rather than clearly defining and comparing the kinetics of each assay and it is clear that detection of bias can change across time.^133^ Finally, and most importantly, we do not yet know which pathways are mediating desired therapeutic effects, and it remains an open question whether or not these can be clearly defined or if the therapeutic effects are mediated through a combination of signaling pathways.

## Abbreviations Used

2-AG: 2-arachidonoylglycerol
CB_1_: cannabinoid receptor 1
CB_2_: cannabinoid receptor 2
CHO: Chinese hamster ovary
CNS: central nervous system
ERK1/2: extracellular signal-regulated kinase 1 or 2
GIRKs: G protein-gated inwardly rectifying potassium channels
GPCR: G protein-coupled receptor
GRKs: G protein receptor kinases
hCB_2_: human CB_2_
JNK: c-Jun-n terminal kinase
KO: knockout
MAP: mitogen-activated protein
mCB_2_: mouse CB_2_
NADA: *N*-arachidonoyl dopamine
PDGF: platelet-derived growth factor
pERK1/2: phosphorylated ERK1/2
PWR: plasmon-waveguide resonance
rCB_1_: rat CB
THC: tetrahydrocannabinol
TRP: transient receptor potential
